# Ozone and childhood respiratory disease in three US cities: evaluation of effect measure modification by neighborhood socioeconomic status using a Bayesian hierarchical approach

**DOI:** 10.1186/s12940-017-0244-2

**Published:** 2017-04-05

**Authors:** Cassandra R. O’ Lenick, Howard H. Chang, Michael R. Kramer, Andrea Winquist, James A. Mulholland, Mariel D. Friberg, Stefanie Ebelt Sarnat

**Affiliations:** 1 Department of Environmental Health, Rollins School of Public Health, Emory University, Second Floor, Claudia Nance Rollins Building, Rm. 2030 B, 1518 Clifton Road NE, Atlanta, GA 30322 USA; 2 Department of Biostatistics and Bioinformatics, Rollins School of Public Health, Emory University, Atlanta, GA USA; 3 Department of Epidemiology, Rollins School of Public Health, Emory University, Atlanta, GA USA; 4 School of Civil and Environmental Engineering, Georgia Institute of Technology, Atlanta, GA USA

**Keywords:** Children’s environmental health, Bayesian, Meta-analysis, Air pollution, Asthma, Socioeconomic status, Environmental epidemiology

## Abstract

**Background:**

Ground-level ozone is a potent airway irritant and a determinant of respiratory morbidity. Susceptibility to the health effects of ambient ozone may be influenced by both intrinsic and extrinsic factors, such as neighborhood socioeconomic status (SES). Questions remain regarding the manner and extent that factors such as SES influence ozone-related health effects, particularly across different study areas.

**Methods:**

Using a 2-stage modeling approach we evaluated neighborhood SES as a modifier of ozone-related pediatric respiratory morbidity in Atlanta, Dallas, & St. Louis. We acquired multi-year data on emergency department (ED) visits among 5–18 year olds with a primary diagnosis of respiratory disease in each city. Daily concentrations of 8-h maximum ambient ozone were estimated for all ZIP Code Tabulation Areas (ZCTA) in each city by fusing observed concentration data from available network monitors with simulations from an emissions-based chemical transport model. In the first stage, we used conditional logistic regression to estimate ZCTA-specific odds ratios (OR) between ozone and respiratory ED visits, controlling for temporal trends and meteorology. In the second stage, we combined ZCTA-level estimates in a Bayesian hierarchical model to assess overall associations and effect modification by neighborhood SES considering categorical and continuous SES indicators (e.g., ZCTA-specific levels of poverty). We estimated ORs and 95% posterior intervals (PI) for a 25 ppb increase in ozone.

**Results:**

The hierarchical model combined effect estimates from 179 ZCTAs in Atlanta, 205 ZCTAs in Dallas, and 151 ZCTAs in St. Louis. The strongest overall association of ozone and pediatric respiratory disease was in Atlanta (OR = 1.08, 95% PI: 1.06, 1.11), followed by Dallas (OR = 1.04, 95% PI: 1.01, 1.07) and St. Louis (OR = 1.03, 95% PI: 0.99, 1.07). Patterns of association across levels of neighborhood SES in each city suggested stronger ORs in low compared to high SES areas, with some evidence of non-linear effect modification.

**Conclusions:**

Results suggest that ozone is associated with pediatric respiratory morbidity in multiple US cities; neighborhood SES may modify this association in a non-linear manner. In each city, children living in low SES environments appear to be especially vulnerable given positive ORs and high underlying rates of respiratory morbidity.

**Electronic supplementary material:**

The online version of this article (doi:10.1186/s12940-017-0244-2) contains supplementary material, which is available to authorized users.

## Background

Ground-level ozone, a criteria pollutant regulated by the US Environmental Protection Agency (USEPA), is a potent airway irritant and well-known determinant of adverse health outcomes, including respiratory morbidity and mortality [1]. Increasing evidence suggests that intrinsic factors (e.g. age, sex, genetics), extrinsic factors (e.g. low socioeconomic status), and differential exposure among populations may potentiate susceptibility to the health effects of ambient ozone [2]. However, questions remain as to the degree of influence these factors exert on ozone-related health effects [3].

Intrinsically, children are considered more vulnerable than adults to the health effects of ozone due to their higher ventilation rates, a developing respiratory system, and time activity patterns that generally increase their exposures to ambient ozone. Concomitantly, physiological differences in airway structure and function cause greater doses of pollutants to be delivered into airways and predispose children to airway inflammation and obstruction [4–6]. Extrinsically, low socioeconomic status (SES) may exacerbate vulnerabilities among children through greater exposure to indoor and outdoor air pollutants, greater psychosocial stress associated with their home or neighborhood environments, and reduced access to vital resources including nutritious food and adequate health care [7–9]. However, findings to date have not conclusively identified SES as a modifier of ozone-related respiratory disease [2, 3]. Results from studies investigating modification of acute air pollution-health risk by neighborhood socioeconomic environments have been particularly inconsistent, reporting weak or contradictory results [10–27]. Among these studies, conclusions about effect modification by neighborhood SES differed depending on indicator choice within in the same study, [11, 15, 24, 25, 27] and differed between study locations even when the same neighborhood SES indicators were used [10, 11, 15, 26]. These observed incongruences call into question whether findings from individual studies, often conducted in single cities or communities, can be generalized.

Previous findings from our research team in Atlanta identified robust associations between ground level ozone and pediatric respiratory health outcomes [27–33]. Analyses examining effect modification of ozone-related pediatric asthma ED visits by neighborhood-level SES suggested non-linear patterns of effect modification by neighborhood SES in Atlanta; for example, in some analyses we observed stronger associations between ozone and pediatric asthma ED visits in the highest and lowest SES strata and weaker associations in middle SES strata [27]. This pattern of effect modification could be partially responsible for the null and unanticipated patterns observed in previous studies. We also found that patterns of effect modification differed depending on our choice of SES indicator and choice of stratification criteria (e.g. median values versus quartile values). However, the generalizability of these findings to other study areas or other respiratory health outcomes has not been established.

Several studies have utilized Bayesian hierarchical models to explore associations between air pollution and adverse health outcomes across multiple study locations, in a computationally efficient manner [34–38]. Furthermore, analyzing multicity data using Bayesian hierarchical models allows for assessment of factors that may help to explain between-location heterogeneity and ultimately ascertain population-level vulnerability factors [34, 35]. Here, we use a two-stage Bayesian hierarchal approach to examine effect modification of ozone-related pediatric respiratory disease by categorical and continuous measures of neighborhood SES in three diverse cities (Atlanta, Dallas, and St. Louis). By applying a consistent analytic approach we assess the generalizability of associations between ozone and pediatric respiratory disease across study areas and evaluate whether patterns of effect modification differ by city.

## Methods

### Emergency department visit data

Multi-year ED visit data were collected from three diverse study locations, which included the metropolitan areas of Atlanta, Dallas, and St. Louis. These data have been used previously in air pollution health effects investigations [18, 33, 39, 40]. For the current analysis, daily ED visit data were available for 2002–2008 from 41 hospitals in 20-county Atlanta; data through 2004 were collected from individual hospitals directly while 2005–2008 data were collected through the Georgia Hospital Association. Daily ED data were available for 2006–2008 from the Dallas-Fort Worth Hospital Council Foundation for 36 hospitals in the 12-county Dallas metro area. In St. Louis, daily ED data were available for 2002–2007 from the Missouri Hospital Association for 36 hospitals in the 16-county metro area. Daily ED visits for respiratory outcomes (upper respiratory infections, bronchiolitis, pneumonia, asthma, and wheeze) were identified using primary International Classification of Diseases, 9th Revision (ICD-9) codes 460–486, 493, 786.07. We restricted our analyses to the pediatric population (5–18 years old) and to patients with a residential ZIP code located wholly or partially in 20-county Atlanta (232 ZIP codes), 12-county Dallas (271 ZIP codes), or 16-county St. Louis (264 ZIP codes). The Emory University Institutional Review Board approved this study and granted exemption from informed consent requirements.

To create spatial scales compatible with air quality and census-based data, each ZIP code in the ED visit database was assigned to a 2010 Zip Code Tabulation Area (ZCTA, Census Bureau boundaries, created from census blocks to approximate ZIP codes). Assignments were accomplished by matching each ZIP code to a 2010 ZCTA based on 5-digit Census ID numbers. ZIP code change reports helped facilitate ZCTA assignments for ZIP codes that were altered or eliminated during the study period. ZCTAs that were classified as businesses or university campuses were excluded from the study. The resulting study areas included 191 ZCTAs in Atlanta, 253 ZCTAs in Dallas, and 256 ZCTAs in St. Louis. 

### Neighborhood-level socioeconomic data

Estimates of ZCTA-level socioeconomic status (SES) were obtained from the 2000 US Census long form and the American Community Survey (ACS) 5-year (2007–2011) summary file, all normalized to 2010 ZCTA borders (“The Time-Series Research Package”, GeoLytics, Inc., East Brunswick, NJ, 2013). In our analyses, ZCTA boundaries were used to represent neighborhoods of patient residence and yearly values of neighborhood-level (i.e. ZCTA-level) SES were estimated by linear interpolation of Census 2000 and ACS 2007–2011 values. We then averaged the yearly values across the study periods of each city (2002–2008 in Atlanta; 2006–2008 in Dallas, and 2002–2007 in St. Louis) to estimate a mean SES value for each neighborhood. To represent neighborhood-level SES, we chose percentage (%) of the population (≥25 years old) with less than a 12th grade education (% < 12th grade), % of households living below the poverty line (% below poverty), and the Neighborhood Deprivation Index (NDI), a composite index comprised of 8 single indicators of SES (i.e. % household low income (<$30,000), % males not in management, % <12th grade, % of households living below the poverty line, % female headed households, % living in crowding, % households on public assistance, and % unemployed civilian population) that were summarized using principle components analysis [41]. To enable comparison of results across different SES indicators analyses were performed for all indicators of neighborhood SES used in this study (% < 12th grade education, % below poverty, NDI).

### Ambient ozone concentration data

Our study used daily estimates of ambient 8-h maximum ozone for each ZCTA in Atlanta, Dallas, and St. Louis. Daily concentrations of ambient 8-h maximum ozone were estimated by combining observational data from network monitors in each city with pollutant concentration simulations from an emissions-based chemical transport model, the Community Multi-Scale Air Quality version 4.5 (CMAQ) model at 12×12 km grids over Atlanta, Dallas, and St. Louis [42]. Ozone concentrations were estimated for each ZCTA by determining the fraction of a ZCTA’s area within each 12×12 km grid cell and area-weighting the observation-simulation data fusion estimates to get the ZCTA-specific value. Although a 12×12 km grid is a relatively large area to assess exposure to air pollutants, ozone is a spatially homogenous secondary pollutant and concentrations are unlikely to vary substantially over the 12×12km grids used in each city. We specifically chose ambient ozone and our exposure modeling approach to minimize the potential for exposure measurement error in each city. Daily meteorological data were obtained from National Climatic Data Centers at Atlanta Hartsfield International Airport, Dallas/Ft. Worth International Airport, and St. Louis Lambert International Airport.

### Statistical analyses

We applied a two-stage modeling approach to estimate associations between daily ZCTA-specific ozone concentrations and pediatric respiratory ED visits, as well as to evaluate effect modification by neighborhood SES across multiple locations. In the first stage (Stage 1), associations between 3-day moving average (lag days 0–2) ZCTA-specific ozone concentrations and pediatric respiratory disease were estimated for every ZCTA in Atlanta, Dallas, and St. Louis in time-stratified case-crossover analyses using conditional logistic regression, matching on year, month, and day of the week of the ED visit. We chose a 3-day moving average of ozone as our a priori lag structure based on previous work [27, 28, 43]. We included additional control for time-varying factors: indicator variables for season (4-levels), periods of hospital participation and holidays; cubic polynomials for 3-day moving average (lags 0–2) maximum temperature and mean dew point; interaction terms between season and maximum temperature; and a cubic spline on day of year (5° of freedom) to control smoothly for recurrent within-window seasonal trends. The general structure of each Stage 1, ZCTA-specific model was:

1 $$ \begin{array}{l} Logit\;\left[ pr\left( Ykt=1\right)\right]=\upbeta 0+{\displaystyle {\sum}_{k=1}^x}{\zeta}_k{\mathrm{V}}_k+\upbeta (ozonetz)+{\varSigma}_{\mathrm{S}}{\varOmega}_{\mathrm{S}}(seasonts)+{\varSigma}_m{\lambda}_m? m? m\\ {}(DOWtm)+{\varSigma}_{\mathrm{n}}{\nu}_{\mathrm{n}}\left(\mathrm{hosp}\_{\mathrm{period}}_{\mathrm{t}\mathrm{n}}\right) + \mathrm{g}\left(\gamma 1,\dots,\ \gamma \mathrm{n};{\mathrm{t}\mathrm{ime}}_{\mathrm{t}}\right)+{\varSigma}_{\mathrm{q}}{\psi}_{\mathrm{q}}\left({\mathrm{meteorology}}_{\mathrm{t}\mathrm{q}}\right)\end{array} $$

where, Y_kt_ indicates whether person k had the event at time t (1 = event; 0 = no event) and t indexes the event and control days. V_k_ denotes the indicator variables that distinguish the case–control sets for the various individuals, x is the total number of case–control sets, and ζ_k_ denotes parameters specific to the case–control sets (which are not estimated in conditional logistic regression). We defined ozone_tz_, as the ozone exposure for subject k at time t in ZCTA z. Other model covariates included indicator variables for season (4-levels), day of week and holidays (DOW), and indicator variables (hosp_period) for periods of hospital participation during the study period. By design, the case-crossover approach controls for individual time-invariant confounders since case and control days are compared for the same person. We also note that the above model assumes (1) pediatric respiratory disease ED visits for different individuals are independent, conditional on the variables in the model, (2) all confounder effects are ZCTA-specific, and (3) a linear association between ambient ozone concentrations and the log odds of a pediatric respiratory disease ED visit. Using Eq. 1 (Stage 1), we estimated the log odds ratio, ${\widehat{\beta}}_Z,$ of ozone on respiratory disease for ZCTA z, and its estimated variance, ${\widehat{V}}_Z$. Stage 1 models with fewer than 50 total ED visits per ZCTA during the study period did not converge; therefore, these ZCTAs were excluded from the second stage (Stage 2) of our modeling approach.

In Stage 2, we fit two-level Bayesian hierarchical models via the R package ‘TLnise’ with noninformative priors [44]. Similar to a meta-regression analysis, ZCTA-specific effect estimates (log odds ratios, ${\widehat{\beta}}_Z,$) were combined to generate city-specific estimates of the short-term association between ozone and pediatric respiratory ED visits, accounting for (1) uncertainty associated with each ZCTA-specific log odds ratio as measured by its asymptotic standard error, and (2) between-ZCTA variability of the true unobserved ZCTA-specific log odds ratio [35, 45, 46]. Specifically, we fit the following Bayesian hierarchical model in Stage 2 analyses:

2 $$ \begin{array}{c}\hfill {\beta}_z\ \Big|\ {\theta}_z,{\widehat{V}}_z \sim N\left({\theta}_z,{\widehat{V}}_z\right)\hfill \\ {}\hfill {\theta}_z\Big|\ {\alpha}_0,\ \gamma,\ {\tau}^2 \sim N\left({\alpha}_0+{\displaystyle \sum_j}{\gamma}_j{X}_{z j},\ {\tau}^2\right)\hfill \end{array} $$

where,

θ_z_ = the unobserved true log odds ratio in each ZCTA

X_zj_ = ZCTA-specific values of ZCTA-level covariates (j) in ZCTA z

α_0_ = the average log odds ratio for ZCTAs

ϒ_j_ = the change in the log odds ratio for a change in X_zj_

τ^2^ = heterogeneity variance across ZCTAs of the unobserved log odds ratio, θ_z_, unexplained by ZCTA-level characteristics, X_zj_. τ reflects the standard deviation and is the parameter we used to assess whether ZCTA-level characteristics explained heterogeneity in the effect of ozone on pediatric respiratory disease across ZCTAs. Modeling assumptions of the Bayesian Hierarchical meta-regression include: (1) ZCTA-specific coefficients are independent and normally distributed with a common heterogeneity variance; and (2) the effect of ZCTA-level covariates on ozone-related respiratory disease is the same for each city (when pooling data from all three cities).

To estimate overall associations between ozone and pediatric respiratory disease, we used Eq. 2 to fit ‘combined’ meta-regressions which pooled data from all three cities (535 ZCTAs) and included indicator variables for each city, represented by X_zj_ in Eq. 2 [i.e. X_(535 x 3)_ = (X_Atlanta(z)_, X_Dallas(z),_ X_St. Louis(z)_]. When estimating overall associations for each city, we do not include an intercept in the modeling equation. This fitted model is equivalent to one with an intercept and indicator variables for two cities. In secondary analyses, we used Eq. 2 to fit “city-specific” meta-regressions which pooled ZCTA-specific data from each city individually (179 ZCTAs in Atlanta; 205 ZCTAs in Dallas; and 151 ZCTAs in St. Louis).

To examine modification of ozone-related respiratory disease by neighborhood SES, we further included X_zj_ covariates in Eq. 2 that characterized ZCTAs with respect to their SES. In these analyses, ZCTAs of extremely low SES were identified using the following SES indicators: ‘undereducated area (yes/no)’ [≥25% of the population aged at least 25 years with <12th grade education; ‘poverty area status (yes/no)’ (≥20% of households living below the federal poverty line); and ‘above the 90th percentile of the NDI (yes/no)’. We also characterized ZCTAs by continuous values of SES and examined linear and non-linear effect modification through linear, quadratic, and cubic functions of neighborhood SES (indicated by continuous values of % <12th grade education, % below poverty, and the NDI).

For our main effect modification analyses we fit ‘combined’ meta-regressions with the assumption that the effect of neighborhood SES on ozone-related respiratory disease is the same for each city. In combined models, X_zj_ covariates included an intercept, two indicators for city of residence (Dallas and St. Louis), and categorical or continuous ZCTA-level SES. For example, the X_zj_ matrix from a combined meta-regression examining effect modification by linear % below poverty was X_(535 x 4)_ = (1, X_Dallas(z),_ X_St. Louis(z)_, X_%poverty(z)_), where ‘1’ is the intercept and represents a ZCTA in Atlanta with 0% poverty. Consequently, all associations reported from combined models are interpreted as a summary estimate of effect modification by neighborhood SES based on data from three cities. To demonstrate the methods we used to fit the combined meta-regression, we have included an R code and example dataset as Additional files 2 and 3 (example data are not real but are similar in magnitude and structure to the output from the case-crossover analyses in Stage 1). In secondary analyses we assessed deviation from our assumption that the effect of neighborhood SES on ozone-related respiratory disease is the same for each city by fitting separate, ‘city-specific’ meta-regressions, which pooled ZCTA-specific data from each city individually. In doing so, the effect of neighborhood SES on ozone-related respiratory disease was estimated separately for each city.

All associations between ozone and pediatric respiratory disease are reported as odds ratios (OR) and 95% posterior intervals (PI) scaled to a 25 ppb increase in ozone. Model parameter estimates were considered significant if the absolute value of the estimate divided by its posterior standard error was greater than 1.96 (analogous to a Z-score). All analyses were performed using SAS 9.4 (SAS Institute, Cary, NC) and R version 3.2.2 (R Foundation for Statistical Computing, Vienna, Austria).

### Graphical representation of data

To compliment our main analyses, we plotted ZCTA-specific ORs in figures and spatial maps. ZCTA-specific ORs were estimated by linear combination of X_zj_ model coefficients and are interpreted as the estimated “mean” OR for each ZCTA. Estimated mean ZCTA-specific ORs were plotted onto spatial maps to help identify other variables that may be spatially correlated with ZCTA-level SES. It is possible that other variables may explain apparent effect modification by ZCTA-level SES and the mapping exercise was used to help generate hypotheses regarding potential confounders. Spatial maps were generated in ArcGIS® version 10.4.1 (Environmental Systems Research Institute, Redlands, CA, USA, 2015).

## Results

### Three cities characterization

The three study sites assessed in this analysis are large, urban cities located in three distinct US regions: the Southeast (Atlanta), Southwest (Dallas), and Midwest (St. Louis). Table 1 presents descriptive statistics for each study site including mean temperature, number of ozone monitors, ozone concentration, and socioeconomic composition of the population.

**Table 1:** Descriptive statistics of temperature, ozone concentrations, and population socioeconomic composition in each city

City	# Counties	# ZCTAs	Temp. (F)	8-hr max Ozone^a^ (ppb)					Socioeconomic Composition	
				# Monitors	Mean (SD)	Min	Max	IQR	% <12th Grade Mean (SD)	% Below Poverty Mean (SD
Atlanta	20	191	63.1	12	42.2 (17.3)	2.21	125	26.0	15.7 (8.10)	13.1 (7.84)
Dallas	12	253	68.8	19	42.0 (14.6)	2.23	118	19.7	17.5 (12.1)	14.0 (9.75)
St. Louis	16	256	57.9	18	40.0 (17.3)	0.15	115	25.4	15.9 (8.14)	12.5 (9.45)

^a^ Daily ZCTA-specific concentrations of ambient 8-h maximum ozone. Mean, SD, min., max., and IQR are summarized across days and ZCTAs

Abbreviations: % <12th grade, percentage of the adult population (≥25 years old) with less than a 12th grade education; % below poverty, percentage of households living below the Federal Poverty Line; #, number; *IQR* interquartile range, *Max* maximum, *Min* minimum, *ppb* parts per billion, *SD* standard deviation; *Temp* average daily mean temperature in degrees Fahrenheit (F); *ZCTA* Zip Code Tabulation Area

Daily mean temperatures during the study period were on average higher in Dallas (68.8 F) compared to Atlanta (63.1 F) and St. Louis (57.9 F). On average, Atlanta and Dallas had slightly greater daily concentrations of ozone across their respective study periods (42.2 and 42.0 ppb) compared to St. Louis (40.0 ppb). With regard to socioeconomic composition, Dallas had the highest mean values of % below poverty (14.0%) and % <12th grade education (17.5%) across ZCTAs, indicative of lower SES neighborhoods, on average, in Dallas compared to Atlanta and St. Louis. Additional file 1: Figure S1 presents additional summary statistics and density distribution plots of % <12th grade, % below poverty, and the NDI for each city. Note NDI values were standardized to mean neighborhood deprivation in each city, hence means of 0 and standard deviations of 1 in each city.

### Pediatric respiratory ED visits

**Fig. 1:**
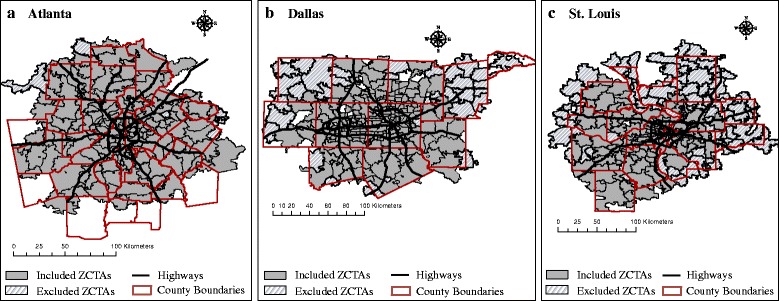
Study area maps for main analyses. Gray areas represent the ZCTAs included in analyses (≥50 respiratory disease ED visits). Hash mark areas represent excluded ZCTAs (<50 respiratory disease ED visits). **a** represents the Atlanta study area; **b** represents the Dallas study area; **c** represents the St. Louis study area. Abbreviations: ED, Emergency Department; ZCTA, ZIP Code Tabulation Area

Our complete ED visit database for respiratory disease among children aged 5–18 years included 211 530 ED visits during the years 2002–2008 in Atlanta, 96 983 ED visits during the years 2006–2008 in Dallas, and 113 285 ED visits during the years 2002–2007 in St. Louis. Due to model convergence issues in the first stage of our analysis, we excluded all ZCTAs that reported fewer than 50 ED counts over their respective study periods. This resulted in the exclusion of 12 ZCTAs in Atlanta, 48 ZCTAs in Dallas, and 105 ZCTAs in St. Louis; these ZCTAs contributed very few ED visits to our overall study and the exclusion of these ZCTAs from analyses resulted in less than 2% of the total number of ED visits from each city to be excluded. Figure 1 presents maps of the included and excluded ZCTAs of the Atlanta, Dallas, and St. Louis study areas. Table 2 summarizes differences in ED data between our complete ED database and the analytical ED database, restricted to data from ZCTAs with at least 50 ED counts.

**Table 2:** Summary of respiratory ED visits^+^ among 5–18 year-olds in Atlanta, Dallas, and St. Louis

	Complete ED Database (data from all ZCTAs)		Analytical ED Database (data from ZCTAs with ≥50 ED visits)					
						ED visits per ZCTA		
City	Number of ZCTAs	ED visit Number^a^	Number of ZCTAs	ED visit Number^b^	% of total ED visits^c^	Min	Mean	Max
Atlanta	191	211 530	179	211 207	99.8%	54	1 180	4 883
Dallas	253	96 983	205	96 108	99.1%	51	469	2 237
St. Louis	256	113 285	151	111 949	98.8%	55	741	5 052

^+^primary diagnosis of respiratory disease (ICD-9 codes 460–486,493,786.07)

^a^total number of respiratory ED visits from all ZCTAs

^b^total number of respiratory ED visits from ZCTAs with ≥ 50 ED visits over the study period of each city

^c^‘total ED visits’ are represented by the ED visit number from the ‘Complete ED Database’

Abbreviations: *ED* Emergency Department, *ICD-9* International Classification of Diseases, 9th Revision, *Max* maximum, *Min* minimum, *ZCTA* Zip Code Tabulation Area

### Epidemiological results: association between ozone and pediatric respiratory disease

The combined meta-regression, which pooled data from all three cities (535 ZCTAs), and city-specific meta-regressions, which pooled ZCTA-specific data from each city individually (179 ZCTAs in Atlanta; 205 ZCTAs in Dallas; and 151 ZCTAs in St. Louis), produced identical overall associations between ozone and pediatric respiratory disease. Ozone exhibited the strongest overall association with pediatric respiratory disease in Atlanta [(OR = 1.08 (95% PI = 1.06, 1.11)], followed by Dallas [OR = 1.04 (95% PI = 1.01, 1.07)] and St. Louis (OR = 1.03 (95% PI = 0.99, 1.07)].

### Epidemiological results: effect measure modification

#### Categorical effect modification

**Fig. 2:**
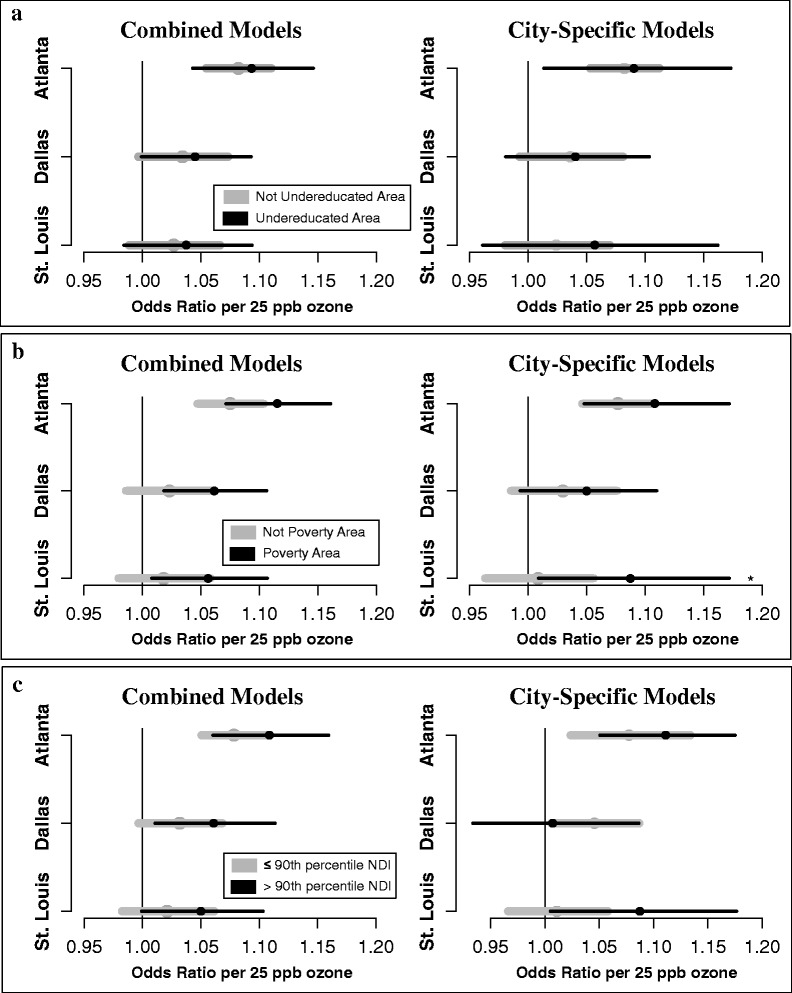
Effect modification by categorical indicators of neighborhood SES using combined and city-specific models. **a**: association between ozone and pediatric respiratory ED visits in undereducated areas (low SES) and non-undereducated areas (high SES). **b**: association between ozone and pediatric respiratory ED visits in poverty areas (low SES) and non-poverty areas (high SES). **c**: association between ozone and pediatric respiratory ED visits in areas above the 90th percentile of the NDI (low SES) and in areas below the 90th percentile (higher SES). Odds ratios and 95% posterior intervals per 25 ppb ozone are presented. Black points and error bars represent ORs and 95% PIs in low SES areas; gray points and bars represent ORs and 95% PIs in areas of higher SES. Undereducated areas: ≥ 25% the adult population (≥25 years old) with less than a 12th grade education. Poverty area: ≥ 20% households living below the Federal Poverty Line. Abbreviations: ED, Emergency Department; NDI, Neighborhood Deprivation Index; SES, socioeconomic status; ZCTA, Zip Code Tabulation Area

Categorical ZCTA-level variables were used in Stage 2 of our modeling approach to assess effect measure modification by neighborhood SES (undereducated area, poverty area, >90th percentile NDI). We did not observe differences in associations between ozone and pediatric respiratory ED visits by undereducated area status when using combined or city-specific models (Fig. 2a). However, when assessing other indicators of neighborhood SES, we observed stronger associations between ozone and pediatric respiratory ED visits in poverty areas for all cities in both the combined and city-specific meta-regressions (Fig. 2b) and stronger associations in areas designated as above the 90th percentile of the NDI with the exception of Dallas in city-specific models (Fig. 2c). These differences in association between SES strata were not statistically significant; however, associations in low SES groups had very wide posterior intervals resulting from very few ZCTAs designated as extremely low SES (Additional file 1: Table S1).

#### Linear and non-linear effect modification

For each city, linear and non-linear effect modification by neighborhood SES was evaluated through the use of linear, quadratic, and cubic functions of % <12th grade education, % below poverty, and the NDI. We present results from combined and city-specific models for estimated ORs across the entire range of neighborhood SES values in each city; interpretations of these results were based on estimated ORs for SES variable values falling between the 2.5th and 97.5th percentiles of neighborhood SES due to data sparseness at the extremes of the SES distributions outside of these bounds.

**Fig. 3:**
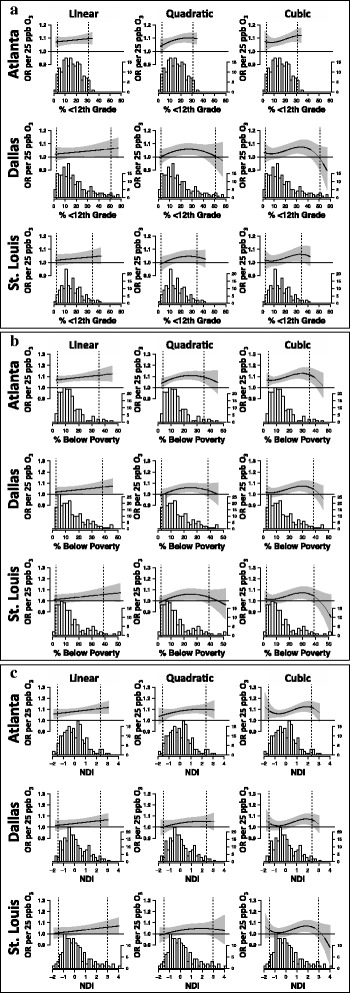
Associations between ozone and pediatric respiratory ED visits by continuous neighborhood SES. Combined meta-regressions were used to examine effect modification of the association between ozone and pediatric respiratory disease by neighborhood SES. Linear, quadratic, and cubic functions of % <12th grade education (**a**), % below poverty (**b**), and the NDI (**c**) were included in combined meta-regressions to examine linear and non-linear effect modification. Solid black lines represent estimated ORs between ozone and pediatric respiratory disease ED visits by ZCTA-specific values of neighborhood SES. Gray polygons represent 95% PIs of the estimated ORs. Histograms below each plot represent the distribution of ZCTA-specific SES values in each city. Dotted black lines represent the 2.5th and 97.5th percentile values of neighborhood SES in each city. The y-axis scale on the right side of each graph represents the frequency count of ZCTAs. Abbreviations: ED, Emergency Department; NDI, Neighborhood Deprivation Index; OR, odds ratio; PI, Posterior Intervals; SES, socioeconomic status; ZCTA, Zip Code Tabulation Area. Plots adapted from Gaspirrini et al., 2015 [53]. R code for plots available at https://github.com/gasparrini/2015_gasparrini_Lancet_Rcodedata [54]

In combined models, estimated ORs tended to increase with decreasing SES, regardless of the continuous function specified in models (linear, quadratic, cubic); this pattern was observed across all SES indicators and in each city (Fig. 3). In Atlanta, robust associations between ozone and pediatric respiratory disease were observed regardless of the socioeconomic environment in which children live. In Dallas and St. Louis, significantly positive estimated ORs were only observed in areas that are characterized as low to very low SES (i.e. above approximately 16% below poverty in Dallas and 20% below poverty in St. Louis). However, in many models specified with quadratic or cubic functions of SES we also observed a decrease in the magnitude of estimated ORs at the lowest extremes of the SES distribution (Fig. 3).

In combined models, we found no evidence of linear effect modification by neighborhood SES, but found some evidence of non-linear effect modification. Specifically, the parameter estimate for the cubic function of the NDI was nearly significant at the 0.05 level (*P* = 0.052, 2-tailed) and the estimated mean ORs varied across NDI levels in a non-linear manner (Fig. 3). Note that in combined models, the relative similarity across cities in linear and non-linear patterns of effect modification reflects the underlying assumption that the effect of neighborhood SES on ozone-related respiratory disease is the same in each city. To assess deviation from this assumption, we also fit city-specific models (Additional file 1: Figure S2). In city-specific analyses, patterns of estimated ORs generally reflected those of the combined models, however, some qualitative differences were observed. The differences between combined and city-specific models were primarily observed when comparing the shape of the nonlinear curve from models fit with quadratic functions of neighborhood SES. For example, when combined and city-specific models were fit with quadratic functions of neighborhood SES, estimated ORs in Dallas followed an inverted U-shape across levels of SES that was not observed in the other cities; however, this pattern was much more dramatic in city-specific models compared to the combined model (Additional file 1 Figure S2).

Although our assessment suggested effect modification by neighborhood SES, inclusion of neighborhood SES in both combined and city-specific models did not substantively explain variability in the unobserved true effect of ozone across ZCTAs as measured by the between-ZCTA heterogeneity parameter, τ (results not shown); these findings imply unexplained heterogeneity across ZCTAs and warrant further inquiry.

#### Spatial mapping and risk visualization

**Fig. 4:**
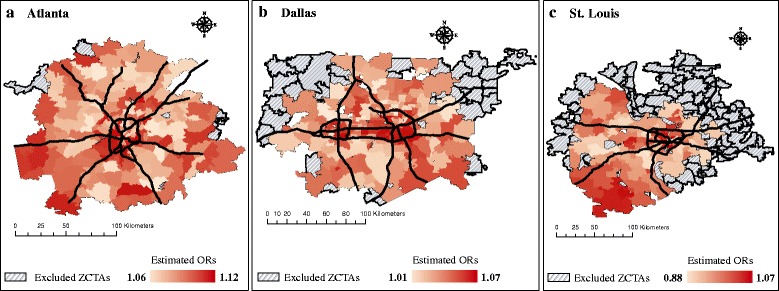
Spatial representation of estimated mean ORs accounting for ZCTA-specific NDI values in each city. In Fig. 4, average ORs between ozone and respiratory disease accounting for ZCTA-specific NDI values were estimated for each ZCTA in Atlanta (**a**), Dallas (**b**), and St. Louis (**c**) using a combined model that included a cubic function of the NDI. Abbreviations: NDI, Neighborhood Deprivation Index; OR, odds ratio; SES, socioeconomic status; ZCTA, ZIP Code Tabulation Area

Spatial mapping, in the context of this study, was used to generate hypotheses about spatial influences and assess potential confounding of apparent effect modification by neighborhood SES. To visually and qualitatively explore spatial patterning, we transferred estimated mean ZCTA-specific ORs from combined models that included cubic functions of the NDI (Fig. 3c) onto spatial maps (Fig. 4). The spatial maps presented in Fig. 4 reveal possible spatial patterning of the ORs and this mapping exercise allowed us to qualitatively assess commonalities among cities and consider possible alternative modifiers of ozone-related respiratory morbidity. For example, ORs appear stronger in areas clustered near urban centers and along major roadways, suggesting common areas of concern in each city. Based on these observations, in secondary analyses we tested whether ZCTAs that included an interstate highway had significantly stronger associations between ozone and respiratory disease; however, we did not find evidence of effect modification by ZCTAs that included an interstate highway (results not shown). Given how we estimated the spatial distribution of ambient ozone (using a regional transport model to interpolate between observations at regulatory ambient monitor sites) we were limited in our ability to detect effect modification associated with nearness to major roadways.

From our mapping exercise we also observed distinct patterns of clustering in each city (e.g. a cluster of high ORs in southwest St. Louis) that may be influencing patterns of effect modification; these differences may be related to patterns of urban development and socio-demographic clustering unique to each city and future analyses could consider performing cluster analyses.

## Discussion

In this study, we assessed the short-term effects of ozone on respiratory ED visits among children in three US cities. We used a 2-stage Bayesian hierarchical approach to examine modification by neighborhood SES and we used information from three cities to improve the representativeness of our results. Our methodology is similar to previous work in this field, but extends that work in two key ways: (1) we specifically focused our meta-regression on ozone-related respiratory disease in the pediatric population, a subpopulation with known sensitivities; and (2) by pooling effects at the ZCTA-level (instead of the city or county-level as is commonly done [34–38]), we were able to quantitatively and qualitatively (through spatial mapping) assess socioeconomic influences at a finer scale resolution than was done previously. Our findings add new insights, and new questions, to the burgeoning knowledge base on neighborhood socioeconomic modifiers of air pollution-health effects.

In overall analyses we observed statistically significant associations between 3-day average concentrations of ozone and pediatric respiratory disease in Atlanta and Dallas. Associations were non-significant in St. Louis, but were similar in magnitude to observed associations in Dallas. These results and their respective magnitudes of association are in line with our previous findings from these cities [18, 29, 33, 40] and with work by others on ozone related respiratory disease [14, 19, 47, 48].

A primary objective of our study was to examine effect modification by neighborhood SES in each city and to evaluate whether patterns of effect modification differed by city. We primarily assessed effect modification through the use of combined meta-regressions that pooled information across ZCTAs in our three cities. By combining information from all ZCTAs we were able to more generally assess the presence of linear and non-linear effect modification across study areas. Another advantage of the combined model approach was greater power to detect effect modification versus city-specific models that had fewer ZCTAs contributing data; however, combined models forced the effect of neighborhood SES on ozone-related respiratory disease to be uniform across all cities. Because neighborhood SES may represent a confluence of extrinsic vulnerability factors and because these factors may differ by city, this is a strong assumption and therefore we also fit city-specific models to assess this assumption. Comparison of results from combined and city-specific models did not yield substantially different interpretations; in fact, patterns of effect modification were largely similar across cities and observed differences could have been due to limited power in city-specific models as well as observed sensitivity of the city-specific models to sparse data at extreme values of neighborhood SES. Therefore, results from combined meta-regressions were used to facilitate interpretations.

In each city, results from combined meta-regressions fit with categorical SES indicators suggested stronger associations between ozone and pediatric respiratory disease in neighborhoods characterized as poverty areas and in neighborhoods above 90th percentile values of the NDI. However, differences between groups were not statistically significant due to wide posterior intervals. Similar patterns were found in Atlanta and St. Louis in previous studies that examined neighborhood SES as a modifier of associations between air pollution and pediatric asthma [18, 27, 49]. When using undereducated area (yes/no) to indicate SES we did not observe differences between strata, suggesting that observed effect modification depended on the way in which neighborhood SES is measured.

In combined meta-regressions fit with continuous values of neighborhood SES, we found some evidence of non-linear patterns of effect modification across levels of SES, particularly for the NDI; overall, these results reflected those observed with categorical indicators of SES in that ORs tended to increase with decreasing neighborhood SES. Our investigation of modification by continuous SES also resulted in the following key observations: (1) we observed robust associations between ozone and pediatric respiratory disease in Atlanta regardless of the socioeconomic environment in which children live (i.e. nearly all ZCTA-specific ORs were significantly positive between the 2.5th and 97.5th percentiles of neighborhood SES). However, in both Dallas and St. Louis, significantly positive associations between ozone and pediatric respiratory disease were only observed in areas that are characterized as low to very low SES (i.e. between the 75th and 95th percentile of neighborhood SES); and (2) in some analyses we observed weak associations in the lowest SES neighborhoods [i.e., neighborhoods at or above the 95th percentile of % below poverty (the extreme right-tail of the SES distribution)].

Non-linear effect modification by continuous neighborhood SES has not been examined previously and findings from this study add to the knowledge base on neighborhood SES as a modifier of air pollution-respiratory disease associations among children. While stronger associations between ozone and respiratory disease have been consistently observed in children compared to adults, [2, 14, 33] the evidence on extrinsic factors (e.g. low socioeconomic status) and their potential to modify ozone-health associations is limited. A recent systematic review by Vinikoor-Imler et al. designates the weight of evidence, regarding neighborhood SES as a modifier as suggestive only, citing “inconsistencies within a discipline” or “lack of coherence across disciplines” as reasons for not being able to make more definitive inferences [2]. Our results suggest potential non-linearity in effect modification, different patterns of effect modification depending on choice of neighborhood SES indicator, and possible spatial patterning of risk. The non-linear patterns and different findings with different SES indicators may account for some of the inconsistencies observed in the studies reviewed by Vinikoor-Imler et al.

Our results also raise additional questions worthy of investigation. For example, why are mean ZCTA-specific ORs weak in the lowest SES neighborhoods? These observations are in stark contrast with our intuition and belief that children from impoverished neighborhoods would be more vulnerable to the respiratory effects of ozone, compared to children living in wealthy neighborhoods. Our study is not designed to answer this question directly, but one possible reason for this observation may be that children living in wealthier neighborhoods have few component causes of air pollution-health effects; therefore, ozone has a substantial relative influence (i.e. a large piece of the ‘causal pie’) on air pollution-health associations [50]. Whereas children in living in lower SES neighborhoods may have a multitude of exposures that could exacerbate respiratory disease, and ozone is only one of many factors (i.e. exposure to ozone constitutes a small piece of the ‘causal pie’).

**Fig. 5:**
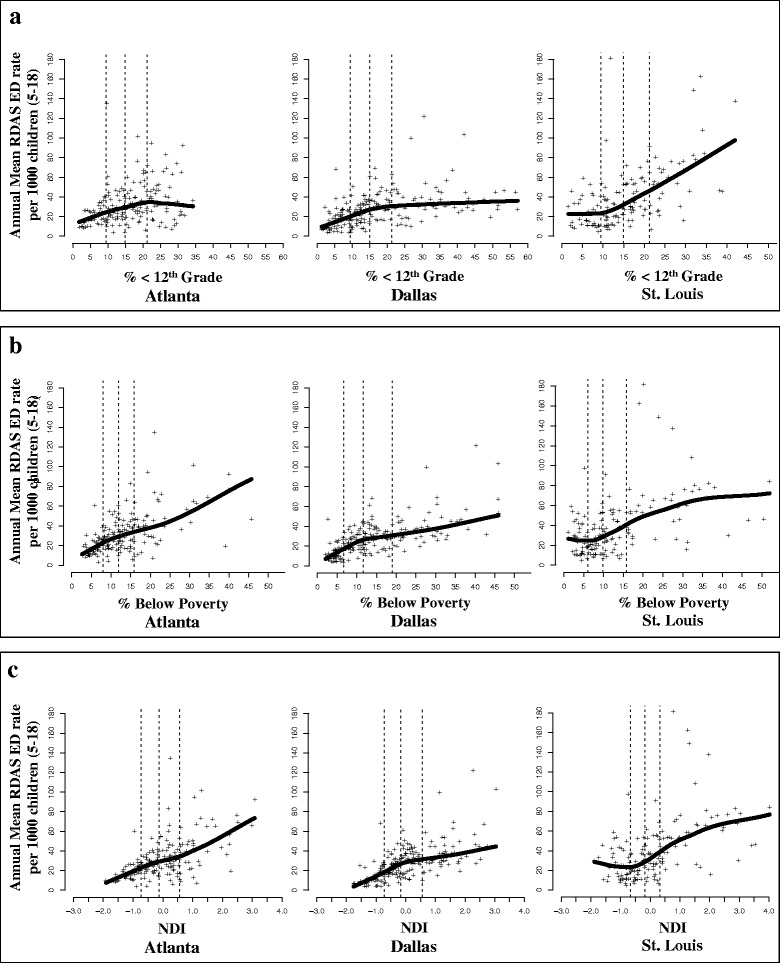
Annual mean ED visit rates by neighborhood SES for each ZCTA in each city. Respiratory disease ED rates are reported per 1000 children (5–18 years old) and were calculated for each ZCTA by dividing the annual total number of respiratory disease ED visits by annual estimates of the 5–18 year old population for each year in the study period. Annual ED Rates were then averaged over the study period of each city. ED visit rates for each ZCTA are represented by the “+” symbol and shown in **a** by percentage (%) of the adult population (≥25 years old) with less than a 12th grade education (% < 12th grade), in **b** by % of households living below the federal poverty line (% below poverty), and in **c** by the Neighborhood Deprivation Index (NDI). The solid black line represents local polynomial regression using weighted least squares to fit a line through the data. The dotted gray lines represent the 1st, 2nd, and 3rd quartile values of each SES indicator. In each panel and city, neighborhood SES decreases from left to right. Abbreviations: ED, Emergency Department; NDI, Neighborhood Deprivation Index; RDAS, respiratory disease ED visits; SES, socioeconomic status; ZCTA, ZIP Code Tabulation Area

Another plausible reason for having observed weaker associations in low SES populations may be due to our use of multiplicative models and the mathematical scale of effect measures. While multiplicative models are used in the vast majority of air pollution-health studies, [3, 51] the true nature of the effect of ozone on ED visits may be additive. In our own data, we observed a marked increase in ED rates from high SES to low SES in each city and for each SES indicator (Fig. 5). Assuming additive effects, low baseline risk could explain stronger relative effects of ozone in the highest SES populations and apparent weaker relative effects in the lowest SES populations [10, 27]. However, in many analyses we observed strong, positive associations in low SES areas, which may reflect supra-additive effects of SES and ozone [27]. While there are methods for estimating additive interaction based on results of multiplicative models (e.g. the Relative Excess Risk due to Interaction (RERI) and the Synergy Index), these methods cannot be straightforwardly applied to our models, and the validity of applying these methods to models with multiple covariates and a continuous exposure is uncertain.

Another potential factor influencing observed associations is complex spatial patterning of respiratory disease risk and socioeconomic status. Our modeling approach enabled us to qualitatively assess similarities and differences in spatial patterning of ozone-health associations across cities by transferring estimated ZCTA-specific ORs onto a spatial canvas to visualize locations of low- and high-risk areas. Findings from this qualitative assessment show that spatial influences are apparent in each city. The observed clustering of health risk and spatial patterning unique to each city may partially account for the patterns of effect modification observed. Future studies can use a similar mapping approach with cluster analysis to assess the degree to which urban development and socio-demographic clustering influence air pollution-health effects.

In our study, inclusion of neighborhood SES in models did not explain heterogeneity in ozone-related pediatric respiratory disease across ZCTAs. There are several limitations that could have contributed to this observation. First, by assessing neighborhood SES effects at the ZCTA level, we assumed that ZCTA boundaries are relevant socioeconomic environments with regards to air pollution vulnerability. However, previous studies using similar methods have only assessed city or county-level effects; [34–38] given that neighborhood SES often varies over smaller spatial scales than counties, our approach, which assessed neighborhood effects at the ZCTA-level, is an improvement over these previous studies. Second, we used neighborhood SES values that were averaged across the study periods to evaluate effect modification of ozone-health associations. While these averages accounted for any shifts in socioeconomic composition that may have occurred during the respective study periods of our three cities, use of these averages in epidemiologic analyses assumed that the SES of all ZCTAs were constant. Due to Dallas’ relatively short study period, we expect this type of exposure misclassification to be less of an issue for Dallas than Atlanta or St. Louis. Third, in our case-crossover models (Stage 1 analyses) we did not include control for other pollutants known to influence respiratory outcomes (e.g. nitrogen dioxide and fine particulate matter). Therefore, our estimated ORs for ozone could include some effects of correlated pollutants. Our decision to only examine health associations with exposure to ozone was based on the fact that ozone is a spatially homogeneous pollutant. In the multi-city context, we were concerned that exposure measurement error might differ in each city due to spatial variation of pollutants within cities. By examining only associations with ozone, we hoped to minimize the effect of such differential exposure measurement error. Nevertheless, we recognize that the associations between ozone and other pollutants could also differ across cities. Finally, although we have large numbers of daily ED visits within each city, power to detect effect modification by socioeconomic factors may have been limited.

## Conclusion

It is well established that ozone is a potent oxidizer and highly toxic to the epithelial cells of the entire respiratory tract. In toxicological studies, acute exposures to ozone induce transient physiological and biochemical changes while chronic exposures lead to cumulative damage or permanent decreases in airway function [52]. Continued efforts to better identify individual- and population-level vulnerabilities, while producing generalizable findings, are imperative.

Our findings suggest that neighborhood-level SES is a factor contributing short-term vulnerability to ozone-related pediatric respiratory morbidity in Atlanta, Dallas, and St. Louis. While nuanced relationships between neighborhood SES and ozone-respiratory health were observed in each city, overall findings were largely generalizable. Synthesizing our results from combined meta-regressions and taking into account the high baseline risk in low SES populations (Fig. 5), we conclude that children living in low SES environments in Atlanta, Dallas, and St. Louis suffer from a higher burden of respiratory disease due to ozone compared to their counterparts living in wealthier SES neighborhoods.

## Additional file

Additional file 1: Figure S1. Distribution and summary statistics for indicators of neighborhood SES in each city. **Table S1.** Number of ED visits and ZCTAs in high and low SES neighborhoods. **Figure S2.** Effect modification of ozone-respiratory disease by continuous values of neighborhood SES in city-specific analyses. (PDF 1175 kb)
Additional file 2: Offers example R code that requires the use of the simulated data in Additional file 3 (log odds ratios, standard errors, and area-level indicators of poverty) to demonstrate how to fit combined Bayesian hierarchical meta-regressions similar to the ones specified in Stage 2 models in O'Lenick et al., 2017.The R code also demonstrates how to make graphs similar to the ones presented in Figure 3 of O'lenick et al., 2017. (XLSX 49.5 kb)
Additional file 3: A simulated dataset that includes variables (log odds ratios, standard errors, and area-level indicators of poverty) for use in the R code in Additional file 2. The variables and the data in this example dataset are simulated, but are representative (similar in magnitude and structure) to outputs from the ZIP Code Tabluation Area (ZCTA)-specific case-crossover models described in Stage 1 models in O'Lenick et al., 2017. (TXT 5.69 kb)

## Abbreviations

ACS: American Community Survey
CMAQ: Community Multi-scale Air Quality
DOW: Day of week
ED: emergency department
EPA: U.S. Environmental Protection Agency
ICD-9: International Classification of Diseases, 9th Revision
NDI: Neighborhood Deprivation Index
OR: Odds ratio
PI: Posterior interval
SES: Socioeconomic status
ZCTA: ZIP Code Tabulation Area

## References

[1] U.S. Environmental Protection Agency. Final Report: Integrated Science Assessment of Ozone and Related Photochemical Oxidants. Washington, DC: U.S. Environmental Protection Agency; 2013. publication no. EPA/600/R-10/076 F) (U.S. Environmental Protection Agency).

[2] Vinikoor-Imler LC, Owens EO, Nichols JL, Ross M, Brown JS, Sacks JD (2014). Evaluating potential response-modifying factors for associations between ozone and health outcomes: a weight-of-evidence approach. Environ Health Perspect.

[3] Bell ML, Zanobetti A, Dominici F (2014). Who is more affected by ozone pollution? a systematic review and meta-analysis. Am J Epidemiol.

[4] Bateson TF, Schwartz J (2007). Children’s response to Air pollutants. J Toxicol Environ Health A.

[5] Makri A, Stilianakis NI (2008). Vulnerability to air pollution health effects. Int J Hyg Environ Health.

[6] Klepeis NE, Nelson WC, Ott WR, Robinson JP, Tsang AM, Switzer P, Behar JV, Hern SC, Engelmann WH (2001). The National Human Activity Pattern Survey (NHAPS): a resource for assessing exposure to environmental pollutants. J Expo Anal Environ Epidemiol.

[7] Adler NE, Newman K (2002). Socioeconomic disparities in health: pathways and policies. Health Aff.

[8] Bernard P, Charafeddine R, Frohlich KL, Daniel M, Kestens Y, Potvin L (2007). Health inequalities and place: a theoretical conception of neighbourhood. Soc Sci Med.

[9] Krieger N, Williams DR, Moss NE (1997). Measuring social class in US public health research: concepts, methodologies, and guidelines. Annu Rev Public Health.

[10] Burra TA, Moineddin R, Agha MM, Glazier RH (2009). Social disadvantage, air pollution, and asthma physician visits in Toronto, Canada. Environ Res.

[11] Delfino RJ, Chang J, Wu J, Ren C, Tjoa T, Nickerson B, Cooper D, Gillen DL (2009). Repeated hospital encounters for asthma in children and exposure to traffic-related air pollution near the home. Ann. Allergy Asthma Immunol..

[12] Laurent O, Pedrono G, Segala C, Filleul L, Havard S, Deguen S, Schillinger C, Riviere E, Bard D (2008). Air pollution, asthma attacks, and socioeconomic deprivation: a small-area case-crossover study. Am J Epidemiol.

[13] Lin M, Chen Y, Villeneuve PJ, Burnett RT, Lemyre L, Hertzman C, McGrail KM, Krewski D (2004). Gaseous air pollutants and asthma hospitalization of children with low household income in Vancouver, British Columbia, Canada. Am J Epidemiol.

[14] Sacks JD, Rappold AG, Davis JA, Richardson DB, Waller AE, Luben TJ (2014). Influence of urbanicity and county characteristics on the association between ozone and asthma emergency department visits in North Carolina. Environ Health Perspect.

[15] Yap P-S, Gilbreath S, Garcia C, Jareen N, Goodrich B (2013). The influence of socioeconomic markers on the association between fine particulate matter and hospital admissions for respiratory conditions among children. Am J Public Health.

[16] Laurent O, Pedrono G, Filleul L, Segala C, Lefranc A, Schillinger C, Riviere E, Bard D (2009). Influence of socioeconomic deprivation on the relation between Air pollution and beta-agonist sales for asthma. Chest.

[17] Norris G, YoungPong SN, Koenig JQ, Larson TV, Sheppard L, Stout JW (1999). An association between fine particles and asthma emergency department visits for children in Seattle. Environ Health Perspect.

[18] Winquist A, Klein M, Tolbert P, Flanders WD, Hess J, Sarnat SE (2012). Comparison of emergency department and hospital admissions data for air pollution time-series studies. Environ Health.

[19] Yang Q, Chen Y, Shi Y, Burnett RT, McGrail KM, Krewski D (2003). Association between ozone and respiratory admissions among children and the elderly in Vancouver, Canada. Inhal Toxicol.

[20] Kim SY, O’Neill MS, Lee JT, Cho Y, Kim J, Kim H (2007). Air pollution, socioeconomic position, and emergency hospital visits for asthma in Seoul, Korea. Int Arch Occup Environ Health.

[21] Lee JT, Son JY, Kim H, Kim SY (2006). Effect of air pollution on asthma-related hospital admissions for children by socioeconomic status associated with area of residence. Arch. Environ. Occup. Health.

[22] Neidell MJ (2004). Air pollution, health, and socio-economic status: the effect of outdoor air quality on childhood asthma. J Health Econ.

[23] Sarnat SE, Sarnat JA, Mulholland J, Isakov V, Ozkaynak H, Chang HH, Klein M, Tolbert PE (2013). Application of alternative spatiotemporal metrics of ambient air pollution exposure in a time-series epidemiological study in Atlanta. J Expo Sci Environ Epidemiol.

[24] Shmool JL, Kubzansky LD, Newman OD, Spengler J, Shepard P, Clougherty JE (2014). Social stressors and air pollution across New York City communities: a spatial approach for assessing correlations among multiple exposures. Environ Health.

[25] Wilhelm M, Qian L, Ritz B (2009). Outdoor air pollution, family and neighborhood environment, and asthma in LA FANS children. Health Place.

[26] Lin S, Bell EM, Liu W, Walker RJ, Kim NK, Hwang SA. Ambient ozone concentration and hospital admissions due to childhood respiratory diseases in New York State, 1991–2001. Environ Res 2008 10.1016/j.envres.2008.06.007 18656858

[27] O’Lenick CR, Winquist A, Mulholland JA, Friberg MD, Chang HH, Kramer MR, Darrow LA, Sarnat SE (2017). Assessment of neighbourhood-level socioeconomic status as a modifier of air pollution–asthma associations among children in Atlanta. J Epidemiol Community Health.

[28] Strickland MJ, Darrow LA, Klein M, Flanders WD, Sarnat JA, Waller LA, Sarnat SE, Mulholland JA, Tolbert PE (2010). Short-term associations between ambient air pollutants and pediatric asthma emergency department visits. Am J Respir Crit Care Med.

[29] Tolbert PE, Mulholland JA, MacIntosh DL, Xu F, Daniels D, Devine OJ, Carlin BP, Klein M, Dorley J, Butler AJ (2000). Air quality and pediatric emergency room visits for asthma in Atlanta, Georgia. USA Am J Epidemiol.

[30] Strickland MJ, Klein M, Flanders WD, Chang HH, Mulholland JA, Tolbert PE, Darrow LA (2014). Modification of the effect of ambient air pollution on pediatric asthma emergency visits: susceptible subpopulations. Epidemiology.

[31] Peel JL, Tolbert PE, Klein M, Metzger KB, Flanders WD, Todd K, Mulholland JA, Ryan PB, Frumkin H (2005). Ambient air pollution and respiratory emergency department visits. Epidemiology.

[32] Darrow LA, Klein M, Flanders WD, Mulholland JA, Tolbert PE, Strickland MJ (2014). Air pollution and acute respiratory infections among children 0–4 years of Age: an 18-year time-series study. Am J Epidemiol.

[33] Alhanti BA, Chang HH, Winquist A, Mulholland JA, Darrow LA, Sarnat SE (2016). Ambient air pollution and emergency department visits for asthma: a multi-city assessment of effect modification by age. J Expo Sci Environ Epidemiol.

[34] Bell ML, Dominici F (2008). Effect modification by community characteristics on the short-term effects of ozone exposure and mortality in 98 US communities. Am J Epidemiol.

[35] Dominici F, Samet JM, Zeger SL (2000). Combining evidence on air pollution and daily mortality from the 20 largest US cities: a hierarchical modelling strategy. J. R. Stat. Soc. A. Stat. Soc..

[36] Levy JI, Diez D, Dou Y, Barr CD, Dominici F (2012). A meta-analysis and multisite time-series analysis of the differential toxicity of major fine particulate matter constituents. Am J Epidemiol.

[37] Chen R, Kan H, Chen B, Huang W, Bai Z, Song G, Pan G, Group CC (2012). Association of particulate air pollution with daily mortality: the China Air Pollution and Health Effects Study. Am J Epidemiol.

[38] Peng RD, Bell ML, Geyh AS, McDermott A, Zeger SL, Samet JM, Dominici F (2009). Emergency admissions for cardiovascular and respiratory diseases and the chemical composition of fine particle air pollution. Environ Health Perspect.

[39] Sarnat SE, Winquist A, Schauer JJ, Turner JR, Sarnat JA. Fine Particulate Matter Components and Emergency Department Visits for Cardiovascular and Respiratory Diseases in the St. Louis, Missouri-Illinois, Metropolitan Area. Environ Health Perspect. 2015;123:437–44. 10.1289/ehp.1307776 PMC4421761 25575028

[40] Winquist A, Kirrane E, Klein M, Strickland M, Darrow LA, Sarnat SE, Gass K, Mulholland J, Russell A, Tolbert P (2014). Joint effects of ambient Air pollutants on pediatric asthma emergency department visits in Atlanta, 1998–2004. Epidemiology.

[41] Messer LC, Laraia BA, Kaufman JS, Eyster J, Holzman C, Culhane J, Elo I, Burke JG, O’Campo P (2006). The development of a standardized neighborhood deprivation index. J Urban Health.

[42] Friberg MD, Zhai X, Holmes HA, Chang HH, Strickland MJ, Sarnat SE, Tolbert PE, Russell AG, Mulholland JA (2016). Method for fusing observational data and chemical transport model simulations to estimate spatiotemporally resolved ambient Air pollution. Environ Sci Technol.

[43] Strickland MJ, Darrow LA, Mulholland JA, Klein M, Flanders WD, Winquist A, Tolbert PE (2011). Implications of different approaches for characterizing ambient air pollutant concentrations within the urban airshed for time-series studies and health benefits analyses. Environ Health.

[44] Everson PJ, Morris CN (2000). Inference for multivariate normal hierarchical models. J R Stat Soc Ser B (Stat Methodol).

[45] Peng RD, Chang HH, Bell ML (2008). Coarse particulate matter air pollution and hospital admissions for cardiovascular and respiratory diseases among medicare patients. JAMA.

[46] Dominici F, Peng RD, Bell ML, Pham L, McDermott A, Zeger SL, Samet JM. Fine particulate air pollution and hospital admission for cardiovascular and respiratory diseases. JAMA 2006; 295(10):popost1127-1134. 10.1001/jama.295.10.1127 PMC3543154 16522832

[47] Villeneuve PJ, Chen L, Rowe BH, Coates F (2007). Outdoor air pollution and emergency department visits for asthma among children and adults: a case-crossover study in northern Alberta, Canada. Environ Health.

[48] Choi M, Curriero FC, Johantgen M, Mills ME, Sattler B, Lipscomb J (2011). Association between ozone and emergency department visits: an ecological study. Int J Environ Health Res.

[49] Sarnat SE, Klein M, Sarnat JA, Flanders WD, Waller LA, Mulholland JA, Russell AG, Tolbert PE (2010). An examination of exposure measurement error from air pollutant spatial variability in time-series studies. J Expo Sci Environ Epidemiol.

[50] Rothman KJ (1976). CAUSES. Am J Epidemiol.

[51] Bell ML, Zanobetti A, Dominici F (2013). Evidence on vulnerability and susceptibility to health risks associated with short-term exposure to particulate matter: a systematic review and meta-analysis. Am J Epidemiol.

[52] Curtis D. Klaassen, Louis J. Casarett, Doull J. Casarett and Doull's toxicology : the basic science of poisons, 8th edn: New York : McGraw-Hill Education, c2013.

[53] Gasparrini A, Guo Y, Hashizume M, Lavigne E, Zanobetti A, Schwartz J, Tobias A, Tong S, Rocklöv J, Forsberg B (2015). Mortality risk attributable to high and low ambient temperature: a multicountry observational study. Lancet.

[54] Gasparrini A. Supplementary data and R-code for “Mortality risk attributable to high and low ambient temperature: a multicountry observational study”. 2015. [https://github.com/gasparrini/2015_gasparrini_Lancet_Rcodedata]. Accessed 3 Feb 2016. 10.1016/S0140-6736(14)62114-0 PMC4521077 26003380

