# Erratum to: Ozone and childhood respiratory disease in three US cities: evaluation of effect measure modification by neighborhood socioeconomic status using a Bayesian hierarchical approach

**DOI:** 10.1186/s12940-017-0275-8

**Published:** 2017-06-19

**Authors:** Cassandra R. O’ Lenick, Howard H. Chang, Michael R. Kramer, Andrea Winquist, James A. Mulholland, Mariel D. Friberg, Stefanie Ebelt Sarnat

**Affiliations:** 1 Department of Environmental Health, Rollins School of Public Health, Emory University, Second Floor, Claudia Nance Rollins Building, Rm. 2030 B, 1518 Clifton Road NE, Atlanta, GA 30322 USA; 2 Department of Biostatistics and Bioinformatics, Rollins School of Public Health, Emory University, Atlanta, GA USA; 3 Department of Epidemiology, Rollins School of Public Health, Emory O’ Lenick et al. University, Atlanta, GA USA; 4 School of Civil and Environmental Engineering, Georgia Institute of Technology, Atlanta, GA USA

## Erratum

This article [1] has been updated to include an R code and example dataset as Additional files 2 and 3. The R code and example dataset are also shown below.

In addition, the following text has been removed from page 4 of the article:

"To demonstrate the methods we used to fit the combined meta-regression, the online supplement includes example R code with an example dataset (example data are not real but are similar in magnitude and structure to the output from the case-crossover analyses in Stage 1".)

Finally, the following text is shown in the methods section:

To demonstrate the methods we used to fit the combined meta-regression, we have included an R code and example dataset as Additional files 2 and 3 (example data are not real but are similar in magnitude and structure to the output from the case-crossover analyses in Stage 1″.)

## Additional files

Additional file 2: Offers example R code that requires the use of the simulated data in Additional file 3 (log odds ratios, standard errors, and area-level indicators of poverty) to demonstrate how to fit combined Bayesian hierarchical meta-regressions similar to the ones specified in Stage 2 models in O'Lenick et al., 2017 [1].The R code also demonstrates how to make graphs similar to the ones presented in Figure 3 of O'lenick et al., 2017 [1]. (TXT 5 kb)

Additional file 3: A simulated dataset that includes variables (log odds ratios, standard errors, and area-level indicators of poverty) for use in the R code in Additional file 2. The variables and the data in this example dataset are simulated, but are representative (similar in magnitude and structure) to outputs from the ZIP Code Tabluation Area (ZCTA)-specific case-crossover models described in Stage 1 models in O'Lenick et al., 2017 [1]. (XLSX 49 kb)
