# Supplementary material for: Ozone and childhood respiratory disease in three US cities: evaluation of effect measure modification by neighborhood socioeconomic status using a Bayesian hierarchical approach
Source: Environ Health. 2017 Apr 5;16:36. doi: 10.1186/s12940-017-0244-2 (PMC5382444; doi:10.1186/s12940-017-0244-2)
Supplement: Additional file 1: Figure S1. — Distribution and summary statistics for indicators of neighborhood SES in each city. Table S1. Number of ED visits and ZCTAs in high and low SES neighborhoods. Figure S2. Effect modification of ozone-respiratory disease by continuous values of neighborhood SES in city-specific analyses. (PDF 1175 kb) [file 12940_2017_244_MOESM1_ESM.pdf]

Supplemental Material

Ozone and childhood respiratory disease in three US cities: evaluation of effect measure modification by neighborhood socioeconomic status using a Bayesian hierarchal approach

Cassandra R. O'Lenick, Howard H. Chang, Michael R. Kramer, Andrea Winquist, James A. Mulholland, Mariel D. Friberg, Stefanie Ebelt Sarnat

Contents

Supplemental Figure S1: Distribution and summary statistics for indicators of neighborhood SES in each city 2

Supplemental Table S1: Number of ED visits and ZCTAs in high and low SES neighborhoods 3

Supplemental Figure S2: Effect modification of ozone-respiratory disease by continuous values of neighborhood SES in city-specific analyses 4

References 5

Supplemental Figure S1. Distribution and summary statistics of ZCTA-level SES in each city. Descriptive results include data from the complete ED database (data from all ZCTAs). Abbreviations: % <12th grade, percentage of the adult population (≥25 years old) with less than a 12th grade education; % below poverty, percentage of households living below the Federal Poverty Line; Max, maximum; Min, minimum; n miss., number missing; NDI, Neighborhood Deprivation Index; p25, 25th percentile; p50, 50th percentile (median), p75, 75th percentile; SD, standard deviation; SES, socioeconomic status, ZCTA, Zip Code Tabulation Area.

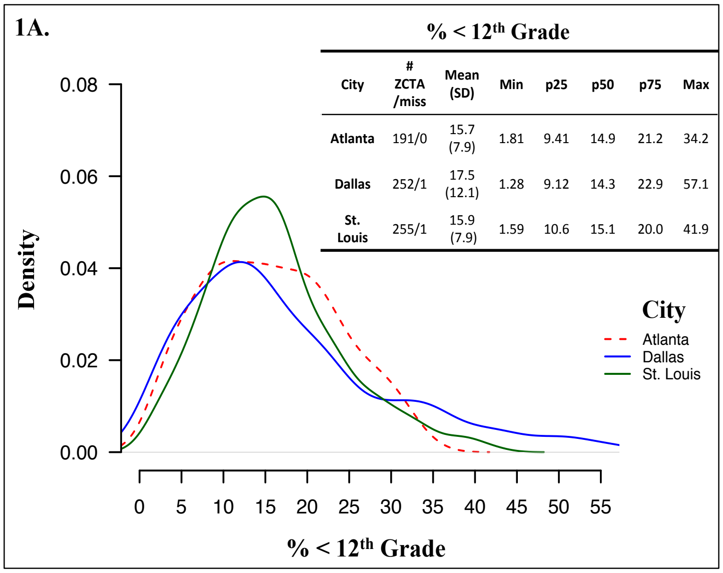

% < 12th Grade

City	# ZCTA /miss	Mean (SD)	Min	p25	p50	p75	Max
Atlanta	191/0	15.7 (7.9)	1.81	9.41	14.9	21.2	34.2
Dallas	252/1	17.5 (12.1)	1.28	9.12	14.3	22.9	57.1
St. Louis	255/1	15.9 (7.9)	1.59	10.6	15.1	20.0	41.9

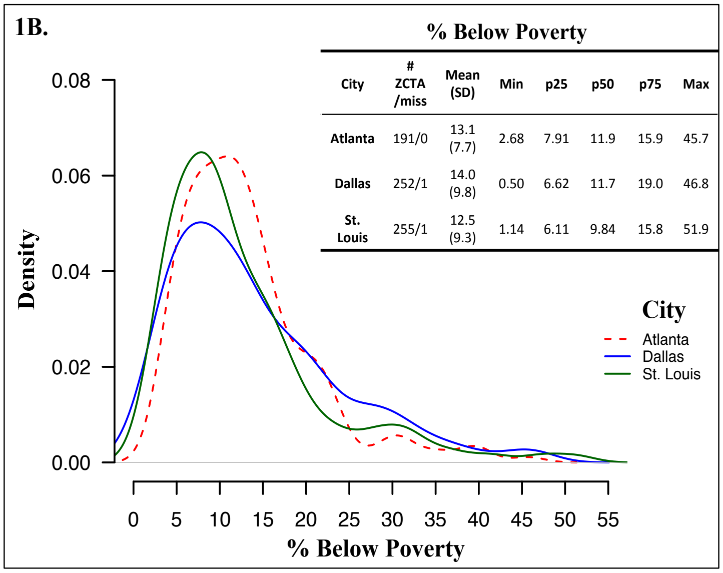

% Below Poverty

City	# ZCTA /miss	Mean (SD)	Min	p25	p50	p75	Max
Atlanta	191/0	13.1 (7.7)	2.68	7.91	11.9	15.9	45.7
Dallas	252/1	14.0 (9.8)	0.50	6.62	11.7	19.0	46.8
St. Louis	255/1	12.5 (9.3)	1.14	6.11	9.84	15.8	51.9

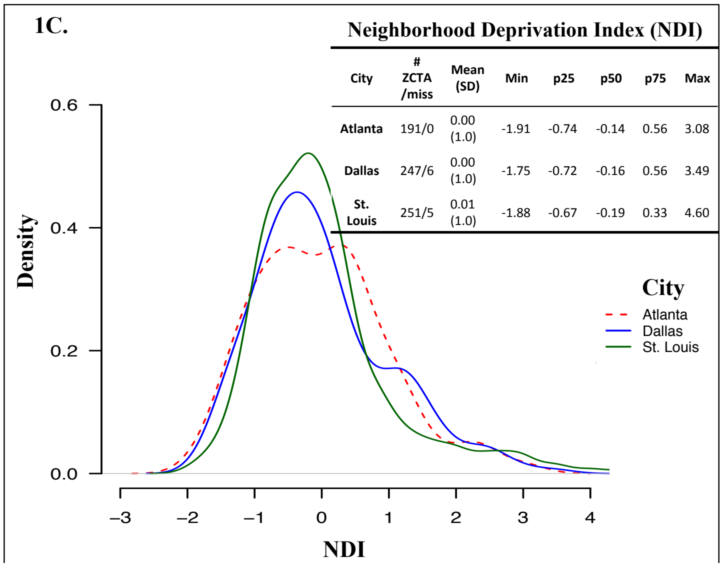

Neighborhood Deprivation Index (NDI)

City	# ZCTA /miss	Mean (SD)	Min	p25	p50	p75	Max
Atlanta	191/0	0.00 (1.0)	-1.91	-0.74	-0.14	0.56	3.08
Dallas	247/6	0.00 (1.0)	-1.75	-0.72	-0.16	0.56	3.49
St. Louis	251/5	0.01 (1.0)	-1.88	-0.67	-0.19	0.33	4.60

Supplemental Table S1. Number of respiratory ED visits and number of ZCTAs in high and low SES neighborhoods[a]

City	Metric	Total #	Undereducated Area (UA) Status		Poverty Area (PA) Status		Deprivation Area Status (≤ or > 90th %tile NDI)	
			Not UA (high SES)	UA (low SES)	Not PA (high SES)	PA (low SES)	≤ 90th %tile NDI (high SES)	> 90th %tile NDI (low SES)
**Atlanta**	#ED visits	211 207	183 156	28 051	163 880	47 327	173 169	38 038
	#ZCTAs	179	155	24	153	26	162	17
**Dallas**	#ED visits	96 108	62 105	34 003	58 181	37 927	76 623	19 485
	#ZCTAs	205	158	47	155	50	182	23
**St. Louis**	#ED visits	111 949	92 261	19 688	80 236	31 713	82 739	29 210
	#ZCTAs	151	131	20	122	29	128	23

[a] Descriptive results limited to data from Analytical ED database (ZCTAs with at least 50 ED counts)

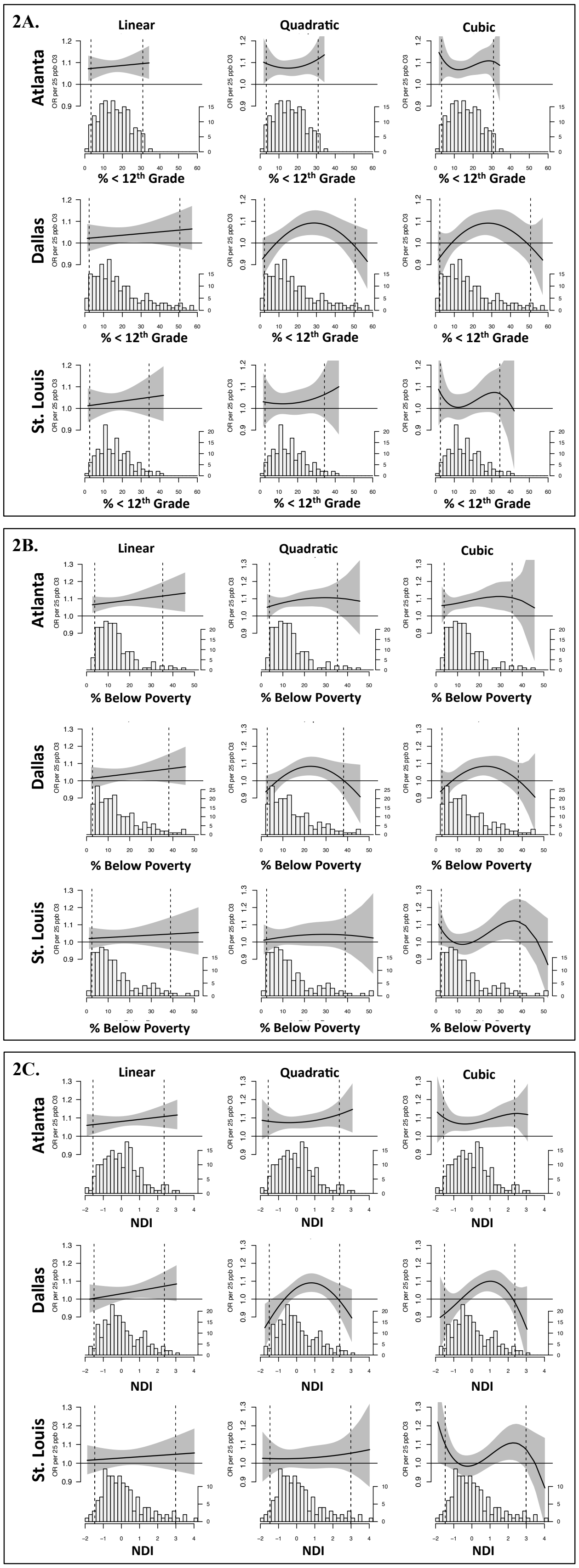

Supplemental Figure S2: Associations between ozone and pediatric respiratory ED visits by continuous neighborhood SES. City-specific meta-regressions were used to examine effect modification of the association between ozone and pediatric respiratory disease by neighborhood SES. Linear, quadratic, and cubic functions of % <12th grade education (2A), % below poverty (2B), and the NDI (2C) were included in city-specific meta-regressions to examine linear and non-linear effect modification. Solid black lines represent estimated ORs between ozone and pediatric respiratory disease ED visits by ZCTA-specific values of neighborhood SES. Gray polygons represent 95% PIs of the estimated ORs. Histograms below each plot represent the distribution of ZCTA-specific SES values in each city. Dotted black lines represent the 2.5th and 97.5th percentile values of neighborhood SES in each city. The y-axis scale on the right side of each graph represents the frequency count of ZCTAs. Plots adapted from R code available in Gaspirrini et al., 2015.[1] R code for plots available at http://www.ag-myresearch.com/lancet2015.html [2] Abbreviations: ED, Emergency Department; NDI, Neighborhood Deprivation Index; OR, odds ratio; PI, Posterior Intervals; SES, socioeconomic status; ZCTA, Zip Code Tabulation Area.

REFERENCES

1. Gasparrini A, Guo Y, Hashizume M, et al. Mortality risk attributable to high and low ambient temperature: A multicountry observational study. *The Lancet* 2015;386(9991):369-75.

2. Gasparrini A. Supplementary data and R code for "Mortality risk attributable to high and low ambient temperature: A multicountry observational study" 2015. Accessed 3 Feb 2016.
